# PSIXPORT: Mobile App for Ecological Momentary Assessment of Psychological Dimensions in Sport Injury

**DOI:** 10.3389/fpsyg.2021.697293

**Published:** 2021-07-27

**Authors:** Luis J. González-Barato, Víctor J. Rubio, José Manuel Hernández, Iván Sánchez-Iglesias

**Affiliations:** ^1^Biological and Health Psychology Department, School of Psychology, University Autonoma, Madrid, Spain; ^2^Psychobiology and Behavioural Sciences' Methodology Department, School of Psychology, University Complutense of Madrid, Madrid, Spain

**Keywords:** sports injuries, ecological momentary assessment, psychological response to injury, mHealth app, rehabilitation process

## Abstract

Retrospective self-reports have been commonly used to assess psychological variables such as feelings, thoughts, or emotions. Nevertheless, this method presents serious limitations to gather accurate information about variables that change over time. The Ecological Momentary Assessment (EMA) approach has been used to deal with some of the limitations these retrospective assessment methods present, and for gathering real-time information about dynamic psychological variables, such as feelings, thoughts, or behaviors. In the sports injury rehabilitation context, athletes' thoughts, feelings, behaviors, and pain perceptions during the rehabilitation process can influence the outcomes of this process. These responses change over different stages of the rehabilitation and taking them into account can help therapists to adapt the rehabilitation process and increasing their effectiveness. With this aim, an EMA mobile app (PSIXPORT) was designed to gather real-time information about severely injured athletes' cognitive appraisals, emotional responses, behaviors, and pain perceptions during their rehabilitation process. The goals of this study were to evaluate Psixport's ability to gather real-time information about injured athletes' psychological responses during the rehabilitation, to test the users' perceived usability of Psixport, and to compare the reliability and differences between real-time data gathered with Psixport and the data gathered through the one-time retrospective method. Twenty-eight severely injured athletes (10 men and 18 women) were assessed using Psixport, a retrospective questionnaire, and the uMARS usability test. Results showed that Psixport can be considered as a good tool to gather information about injured athletes' cognitive appraisals, emotional responses, behaviors, and pain perceptions. Moreover, multiple data assessments gathered with the app showed to be more accurate information about injured athletes' psychological responses than one-time retrospective reports.

## Introduction

Assessing coping strategies from subjects that went through a stressful event involves gathering information about individuals' inner events, such as thoughts or mental images, which can be only provided by the participant (Smith et al., 1999). One-time self-reports have been the prevailing method for assessing such variables (Smith et al., 1999).

However, one-time retrospective assessments could be inaccurate due to different factors, such as subjects' altered recalls based on reconstructive memory processes or incomplete recording of the stressful event (Loftus, 1980; Bradburn et al., 1987; Christianson, 1992; Schwarz and Sudman, 1994). Lack of clear recall of subjects' own thoughts, feelings, or behaviors triggered by or related to those stressful events, could bring participants to base their retrospective reports on the way they generally cope with this type of events (Schwarz and Sudman, 1994; Smith et al., 1999) regardless what they truly feel, though, or behave. That is because authors such as Reis and Wheeler (1991) asserted that gathering information by multiple assessments across time based on diary methodologies could provide more accurate information than one-time retrospective reports.

Ecological Momentary Assessment (EMA) is an assessment approach that allows collecting information about sampling subjects' experiences and behaviors in real-time (or closer to real-time) in their natural environment (Shiffman et al., 2008). Ecological Momentary Assessment approach, compared to retrospective methods, provides multiple advantages such as collecting real-time information about subjects' habits and behaviors from their context (Hicks et al., 2010) or allowing to explore antecedents and consequences after individuals' behaviors appearance (Sala et al., 2017). Consequently, an EMA approach deals with some of the limitations of traditional retrospective methods' such as inaccurate subjects' retrospective answers due to their autobiographical memory biases (Schwarz, 2007).

Moreover, fields such as health or sports demand collecting information about variables that are changing and/or dynamic in nature. Variables such as frequent behaviors, subjective perceptions, or intensity of feelings, change through time and a one-time aggregate gathering prevent investigators from an accurate collection of the variability and complexity such variables present (Schwarz, 2007). For instance, Eich et al. (1985) assessed the maximum, minimum, and current pain levels of chronic pain patients through daily reports and weekly retrospective measures. Comparing retrospective responses with patients' daily diaries responses, they found that retrospective scores were similar to patients' current level of pain at the time the recall was requested, overestimating or underestimating last week's pain. Ecological momentary assessment allows an accurate assessment of such dynamic variables that change through time and cannot be retrospectively assessed as an aggregate score without a significant reduction in variability (Schwarz, 2007).

Ecological momentary assessment approach has been also incorporated to sport psychology research and recent times have shown several studies using such an approach to assess different variables. For instance, Sala et al. (2017) carried out a study analyzing the relationships between daily stress and exercise behaviors in female undergraduate students gathering subjects' exercise behaviors and affective responses to exercise using handheld electronic diaries. Williams et al. (2016) studied the mediation effects of affective responses to exercise on exercise adherence. They collected female college students' stress and exercise measures through four daily assessments across seven days using an automatic EMA telephone system. Moreover, Kim and James (2019) have also used mobile devices to collect information about the subjective well-being perception of college students gathering the fluctuations of their daily responses.

Mobile devices became particularly useful tools for an EMA approach (Kenny et al., 2016). Mobile apps have been used for assessing the relationships between physical and psychological variables such as time-varying associations, momentary behavioral cognitions, and changes in physical activity in adults (Maher et al., 2016), or gathering real-time information of cognitive and physical activity (Wiebe et al., 2016). This is possible nowadays due to almost everyone owns a smartphone and its use it's widely extended (Ltd, 2021) and Mobile Health apps (mHealth apps) use have increased over lasts years (Riley et al., 2011).

When referring to the psychological variables involved in sports injury rehabilitation, the aforementioned changing, and dynamic nature of variables such as individual thoughts, emotions, and behaviors play an important role in the sports injury rehabilitation process (Brewer et al., 2000b). In the sports injury rehabilitation process, athletes experience a range of psychosocial challenges that change during the rehabilitation (Clement et al., 2015). These psychological factors influence athletes' adherence to the rehabilitation contributing to or hampering this process (Brewer et al., 2000a; Almeida et al., 2014; Palmi, 2014).

The Integrated Model of Psychological Response to Sports Injury and Rehabilitation Process (Wiese-Bjornstal et al., 1998), sets athletes' personal and situational factors affect their cognitive appraisals, emotional responses, and behavioral responses during the rehabilitation process (Wiese-Bjornstal, 2014). These responses directly affect athletes' recovery outcomes and return to play process (Wiese-Bjornstal et al., 1998, 2015; Brewer et al., 2000a; Mitchell et al., 2014). Injured athletes' psychological responses to stressors related to sports injuries such as incapacitation thoughts or loss of confidence beliefs, could intensify feelings of frustration (Johnston and Carroll, 1998; Magyar and Duda, 2000), influencing athletes' rehabilitation behaviors such as adherence and conditioning the rehabilitation outcomes (Brewer et al., 2000b). This influencing cycle usually follows this path, but it could go in the opposite direction instead (Wiese-Bjornstal et al., 1998).

In this vein, Wiese-Bjornstal et al. (1998) postulated that assessing athletes' psychosocial responses by repeated measures, tracking the different phases of their rehabilitation and return to play process, must show a most accurate and wider view of the associated risks and their effects across the athlete's rehabilitation journey.

With this aim, an EMA approach mobile app (PSIXPORT) was designed to collect real-time data about injured athletes' psychosocial responses (cognitive appraisals, emotional responses, and behavioral responses) and pain perception across their rehabilitation process according to the Integrated Model of Psychological Response to Sports Injury and Rehabilitation Process (Wiese-Bjornstal et al., 1998).

The present study has three main goals; Firstly, to study the feasibility of Psixport to collect real-time data of injured athletes' responses during their rehabilitation process; Secondly, to test Psixport's usability for subjects, collecting athletes' opinions using the app; Thirdly, to compare the differences between EMA data gathered with Psixport and the data gathered by the retrospective method.

## Materials and Methods

### Participants

Severely injured athletes were recruited from Madrid's (Spain) sports medicine unit, the Regional football Players' Mutual Benefit Society, and different sports clubs. Inclusion criteria were (a) being over 18, (b) having sustained a severe sports injury (ACL tear) that need surgery but have not been operated yet, and (c) having a rehabilitation prescription with a physical therapist after the surgery. The resulting sample was 28 participants, 10 males (35.7%) and 18 women (64.2%) whose mean age was 24.7 (*Mdn* = 24, *SD* = 4.1), who completed Psixport and retrospective questionnaire assessment. Of those 28 subjects, all but one completed the uMARS test. Those severely injured athletes came from multiple sports such as football (Soccer) (*n* = 17; 60.7%), Basketball (*n* = 7; 25%), Indoor football (soccer) (*n* = 1; 3.6%), Frisbee (*n* = 1; 3.6%), Judo (*n* = 1; 3.6%), and Handball (*n* = 1; 3.6%).

This study is part of a wider research project focused on studying the influence of mediation and moderator effects of psychosocial variables on rehabilitation and return to play process of severely injured athletes.

### Instruments and Variables

#### Psixport

A mobile app was designed for assessing athletes' cognitive appraisals, emotional responses, behavioral responses, and pain perception during their rehabilitation process from an EMA approach. Psixport assessment laid on several instruments used for assessing injured athletes' psychological, emotional, behavioral responses, and pain perception, which were adapted to this format. Item responses ranged from 0 to 100 in a continuous Visual Analog Scale, with just the exception of the items regarding whether the athlete has got a rehab session that day and if he/she attended it.

##### Cognitive Appraisals

Evans et al. (2008) developed the Psychological Responses to Sports Injury Inventory (PRSII) to provide a tool that allowed assessing injured athletes' cognitive appraisals regarding their injuries. This test gathers psychological responses resulting on six different dimensions: Devastation, Dispirited, Reorganization, Feeling cheated, Restlessness, and Isolation. Psychological Responses to Sports Injury Inventory was demonstrated to be a valid and reliable test to assess injured athletes' psychological responses to injury (Cronbach's α = 0.86) (Lepley et al., 2018). The six items, one from each dimension, which showed the highest factor loadings were included in Psixport. The items were: Devastation, “I experience a feeling of emptiness. My world has fallen apart”; Dispirited, “I'm lack of motivation”; Reorganization, “I am beginning to feel like myself again”; Feeling Cheated, “I can't help but feel bitter”; Restlessness, “I'm unable to relax, I feel uneasy”; Isolation, “I feel isolated.”

##### Emotional Responses

An adaptation of the picture-oriented Self-Assessment Manikin (SAM) instrument (Lang, 1980; Hodes et al., 1985) assessing two dimensions of emotion; Emotional Valence and Arousal, were used to gather information about athletes' emotional responses during their rehabilitation process. Self-Assessment Manikin was demonstrated to be an effective method to assess feeling states, emotional responses to an event, and their changes over time (Bradley and Lang, 1994). Self-Assessment Manikin was validated as an easy and non-verbal method for assessing affective experiences due to the relationships with the semantic differential methodology (Bradley and Lang, 1994). The two questions included in the app related to emotional responses were “Today I feel… Sad/Happy”; and “Today I feel… Apathetic/Active.”

##### Behavioral Responses

Athletes' compliance with the rehabilitation was the variable selected for assessing injured athletes' behavioral responses. In this case, an adapted version of the “*Sports Injury Rehabilitation Adherence Scale”* (SIRAS) (Brewer et al., 2000a) was used. Sports Injury Rehabilitation Adherence Scale registers athletes' exercise intensity perceived by their therapist during the rehabilitation sessions, the way athletes follow their instructions, and the receptiveness of individuals on rehabilitation changes with three items. An adapted version of SIRAS was designed to gather athletes' self-perceptions instead of therapists' perceptions. Higher scores show higher compliance with the rehabilitation. Sports Injury Rehabilitation Adherence Scale has shown positive correlations with adherence to rehabilitation and exercise completion out of the rehabilitation sessions (Brewer et al., 2002). Moreover, SIRAS has shown internal consistency (α = 0.86) for adherence to rehabilitation sessions across the rehabilitation process (Brewer et al., 1996) and an over 1-week period test-retest reliability coefficient of.65. Four items were adapted and included in the app for assessing athletes' compliance with the rehabilitation process asking them directly: “Did you have rehabilitation session today?” (for this item, the response options were yes/no); “Did you go to the rehabilitation session?” (for this item, the response options were yes/no); “With which intensity did you complete the exercises during today's appointment?”; and “To what extent did you complete the prescribed exercises by yourself?”

##### Pain Perception

Two questions of the “*Universal Pain Assessment Tool”* (UPAT) were included in Wong-Baker's (Wong and Baker, 1988) Faces Pain Rating Scale format to make easier the response choice. This test has provided to be a useful tool for assessing pain levels on athletes (Dugashvili et al., 2017): “Which is the greatest pain you've experienced today due to your injury?” and “How much pain has you experienced today, on average, due to your injury?”

##### Interfaces

Psixport starts with a welcome message, explaining the investigation goals, shows examples about the questions to be answered and the correct way to do so.

Once the athlete joins the study by accepting and signing the informed consent included in the app, the app gathers demographic information such as name, surname, birth date, gender, sport, and competition. Then, subjects must select the date to begin the assessment. This period must start when the athlete attends his/her first rehabilitation session. Likewise, athletes must choose the schedule to complete the daily assessment, which must always be after finishing their regular rehabilitation session. Once completed the daily assessment, Psixport congrats the athlete for the completion and grants him one point.

Furthermore, a four-chart made-up feedback module was included, one for each dimension assessed (cognitive appraisals, emotional responses, behavioral responses, and pain perception) to allow the athlete to check his/her progress in the different areas. Each chart gathers all the scores given by the athlete during his rehabilitation process. The feedback module was included to serve as a motivational factor to improve athletes' commitment and engagement with the app.

#### Retrospective Questionnaire

A retrospective questionnaire (see Appendix) based on Psixport items about the psychosocial variables assessed (cognitive appraisals, emotional responses, behavioral responses, and pain perception) was designed to collect data with a classical retrospective approach to compare the accuracy and quality between Psixport and traditional retrospective collected information. Psixport items were written up retrospectively and presented in a 0–100 point scale format to achieve this purpose.

#### User Version of the Mobile Application Rating Scale

Developed by Stoyanov et al. (2016) the uMARS main goal is to assess mHealth mobile apps quality through users' opinions. This test consists of 20 items including four objective quality subscales (Engagement, Functionality, Aesthetics, and Information quality), one subjective quality subscale, and app perceived impact items.

uMARS items are presented in a five-point Likert scale from “1—Inadequate to 5—Excellent” for assessing the “App Quality Assessment” (Engagement, Functionality, Aesthetics, and Information quality), “1—Strongly disagree to 5—Strongly Agree” items for assessing App Perceived Impact, and with different answering options for Subjective Quality Scale.

uMARS has demonstrated fairly high internal consistency (Cronbach alpha = 0.90) for the full scale and good levels for the subscales (engagement alpha = 0.80; functionality alpha = 0.70; aesthetics alpha = 0.71; information alpha = 0.78; and satisfaction alpha = 0.78), and good levels of internal consistency for 1–2 months test-retest period (0.66) and for 1–3 months period (0.70). This test was used to gather information about users' feedback on Psixport's quality.

### Procedure

After obtaining the University Autonoma of Madrid's IRB approval (CEI-75-1370), we contacted to one of Madrid (Spain) orthopedics clinic specialized in sports medicine, the Madrid's Regional Soccer Association Players' Mutual Benefit Society, and different sports clubs to recruit athletes who fulfill the inclusion criteria.

Participants were informed about the research goals and were provided with a written consent prior to joining the study. No economic compensation was offered to any subject or institution.

In this preliminary phase, athletes downloaded Psixport and answered at least 15 daily assessments about cognitive appraisals, behavioral responses, emotional responses, and pain perception. The initial assessment period was 15 days, giving the option to continue completing the daily assessments after the initial 15-day period requested. Subjects could only complete one assessment per day and must have always been after the prescribed rehabilitation session finished.

Approximately 2 months after completing their last Psixport assessment, subjects were asked to complete a retrospective questionnaire designed to assess the mentioned variables (cognitive appraisals, emotional responses, behavioral responses, and pain perception) from the traditional one-time retrospective approach, and the User version of the Mobile Application Rating Scale (uMARS) to collect information about the designed app quality. Data from the retrospective Psixport test and uMARS test was collect with Qualtrics software, version 03/2020 of Qualtrics. Copyright ©2020 Qualtrics. Qualtrics and all other Qualtrics product or service names are registered trademarks or trademarks of Qualtrics, Provo, UT, USA. https://www.qualtrics.com.

### Data Analysis

We calculated descriptive statistics for participant's age and gender, uMARS objective quality subscales, subjective quality, and perceived impact for Psixport.

The longitudinal records gathered with Psixport were summarized averaging the subject's scores through time, for each item. We calculated descriptive statistics for the items (both the average scores and the scores from the retrospective questionnaire). We used autoregressive (AR) models for analyzing time-series data. Autoregressive models are specific regression models which account for the autocorrelation in time-series data (i.e., the extent to which the dependent variable value depends on past values of itself). These models are consistently used in behavioral research (Crosbie, 1993; Velicer and Colby, 1997; Velicer and Fava, 2003). To graphically show the examples of within-subject, longitudinal scores, and to illustrate the process of forecasting, we used Autoregressive Integrated Moving Average (ARIMA) models (Box and Jenkins, 1970; Glass et al., 1975; McCleary and Hay, 1980; Gottman, 1981) on variables from our dataset, which captures the effects of trend, season, and autocorrelation in time-series data. The first forecasted value for each item was stored as variables, and descriptive statistics were calculated.

The Friedman's test was used to compare the means between Psixport averaged, forecasted, and retrospective scores of 11 out of 14 items (the three remaining items could not be compared, as they were nominal variables), adding *post-hoc* Wilcoxon's tests analyses when needed. Also, we used non-parametric correlation to study the relationship between those three variables.

The significance level was set at α = 0.050. However, to avoid an increase in Type I error for multiple comparisons, the Bonferroni correction was applied in *post-hoc* Wilcoxon's tests and non-parametric *Rho*, setting α at 0.017. All statistical analyses were performed using SPSS 25 (IBM Corp. Released 2017. IBM SPSS Statistics for Windows, Version 25.0. Armonk, NY: IBM Corp.).

## Results

The total number of evaluations gathered with Psixport was 621 from 1,116 possible (summative of the total days of app's use by the participants during their rehabilitation processes), which sets the compliance ratio at 55.7%.

The number of days which participants completed the app ranged from 16 to 98 and the mean average in the number of assessments per subject was 22.2 (*SD* = 10.3), above the 15 initially requested, though not necessarily consecutive. The highest number of assessments completed by an athlete was 58 and the minimum was 15. Moreover, the highest number of consecutive assessments completed for one athlete was 22 and the minimum was 1. Furthermore, the consecutive evaluations' ratio decreased over time until the athlete finally stopped completing the app.

Tables 1, 2 show the descriptive statistics of uMARS objective quality subscales, and subjective quality and perceived impact, respectively.

**Table 1:** uMARS objective subscale scores for Psixport.

**Subscale**	***M* (From 1 to 5)**	***SD***	***Mdn***	***IQR***
Engagement	3.6	0.6	3.63	2.4
Functionality	4.4	0.4	4.33	1.75
Aesthetics	3.8	0.6	3.74	2.34
Information	4.4	0.5	4.39	2.00
App quality	4.1	0.4	4.09	0.80

*N = 27*.

**Table 2:** uMARS subjective quality score and perceived impact for Psixport.

**Subscale**	***M* (From 1 to 5)**	***SD***	***Mdn***	***IQR***
Subjective app quality	3.3	0.6	3.25	2.75
Perceived impact	3.4	0.9	3.33	4.00

*N = 27*.

Table 3 depicts the descriptive statistics and comparison of averaged, forecasted, and retrospective means of Psixport items. On 5 items (out of 11) we did not find mean differences. On one item we found statistical differences between the averaged and forecasted means, but not between any of those and retrospective scores. On five items out of 11 we found statistical differences between the retrospective and both averaged and forecasted means, but no differences between the latter two.

**Table 3:** Psixport items using ecological momentary assessment (EMA) average, EMA forecast, and retrospective measurement.

	**EMA average (1)**		**EMA forecast (2)**		**Retrospective (3)**				**(1–2)**		**(1–3)**		**(2–3)**	
	***M***	***SD***	***M***	***SD***	***M***	***SD***	*X*^2^^*^	***p***	z^*^^*^	***p***	z^**^	***p***	z^**^	***p***
Exercise commitment	76.3	14.1	77.2	19.2	81.4	18.0	5.51	0.064	–	–	–	–	–	–
I feel: active–apathetic	69.8	14.0	70.8	12.9	55.4	24.9	5.89	0.053	–	–	–	–	–	–
I feel: happy–sad	67.9	15.7	69.4	15.1	44.6	27.4	6.89	0.032	−0.94	0.349	−2.70	0.007	−2.98	0.003
Devastation	22.7	18.4	20.0	18.1	42.1	31.3	14.57	0.001	−0.77	0.444	−3.55	0.000	−3.80	0.000
Dispirited	31.5	25.7	28.6	28.3	35.4	25.7	3.46	0.178	–	–	–	–	–	–
Re-organization	53.5	19.0	56.8	19.7	49.3	22.4	6.62	0.036	−2.85	0.004	−1.32	0.187	−1.62	0.106
Feeling cheated	26.8	23.1	24.0	23.9	48.9	28.7	25.93	<0.001	−1.38	0.167	−4.18	0.000	−4.25	0.000
Restlessness	31.7	25.9	27.7	26.4	37.1	31.0	0.80	0.669	–	–	–	–	–	–
Isolation	29.6	25.8	27.0	25.0	45.0	31.2	10.23	0.006	−0.03	0.976	−2.86	0.004	−3.17	0.002
Maximus pain	53.0	22.5	49.6	27.2	75.4	22.2	15.78	<0.001	−0.56	0.574	−3.93	0.000	−4.03	0.000
Pain average	47.7	21.7	45.7	24.5	50.7	19.0	2.79	0.248	–	–	–	–	–	–

*Comparison of means. N = 28. EMA Forecast was estimated with ARIMA models, predicting the next value after the last reported*.

* *Chi-squared based on Friedman's test with df = 2*.

** *Post-hoc Z statistic based on Wilcoxon's test. Corrected α = 0.017*.

Table 4 shows the correlations between averaged, forecasted, and retrospective scores of Psixport items. Most of the correlation values were statistically significant, except for one item on the averaged—retrospective correlations, and four items in the forecasted—retrospective correlations. More interestingly, the effect size was higher for the averaged—forecasted correlations (*R*^2^ ranging from 0.63 to 0.98), and lower for the averaged—retrospective (0.18 to 0.58) and forecasted—retrospective correlations (0.14 to 0.57).

**Table 4:** Correlation of Psixport items scores using ecological momentary assessment (EMA) average, EMA forecast, and retrospective measurement.

	**EMA average—EMA forecast**		**EMA average—retrospective**		**EMA forecast—retrospective**	
	***Rho***	***p***	***Rho***	***p***	***Rho***	***p***
Exercise commitment	0.79	<0.001	0.52	0.005	0.37	0.052
I feel: active–apathetic	0.99	<0.001	0.42	0.024	−0.41	0.029
I feel: happy–sad	0.95	<0.001	0.54	0.003	−0.45	0.018
Devastation	0.86	<0.001	0.70	<0.001	0.59	0.001
Dispirited	0.90	<0.001	0.50	0.006	0.50	0.007
Re-organization	0.94	<0.001	0.54	0.003	0.44	0.019
Feeling cheated	0.87	<0.001	0.76	<0.001	0.61	0.001
Restlessness	0.88	<0.001	0.75	<0.001	0.76	<0.001
Isolation	0.96	<0.001	0.65	<0.001	0.63	<0.001
Maximus pain	0.95	<0.001	0.46	0.002	0.56	0.002
Pain average	0.93	<0.001	0.53	0.004	0.48	0.009

*N = 28. EMA forecast was estimated with ARIMA models, predicting the next value after the last reported. Corrected α = 0.017*.

**Figure 1:**
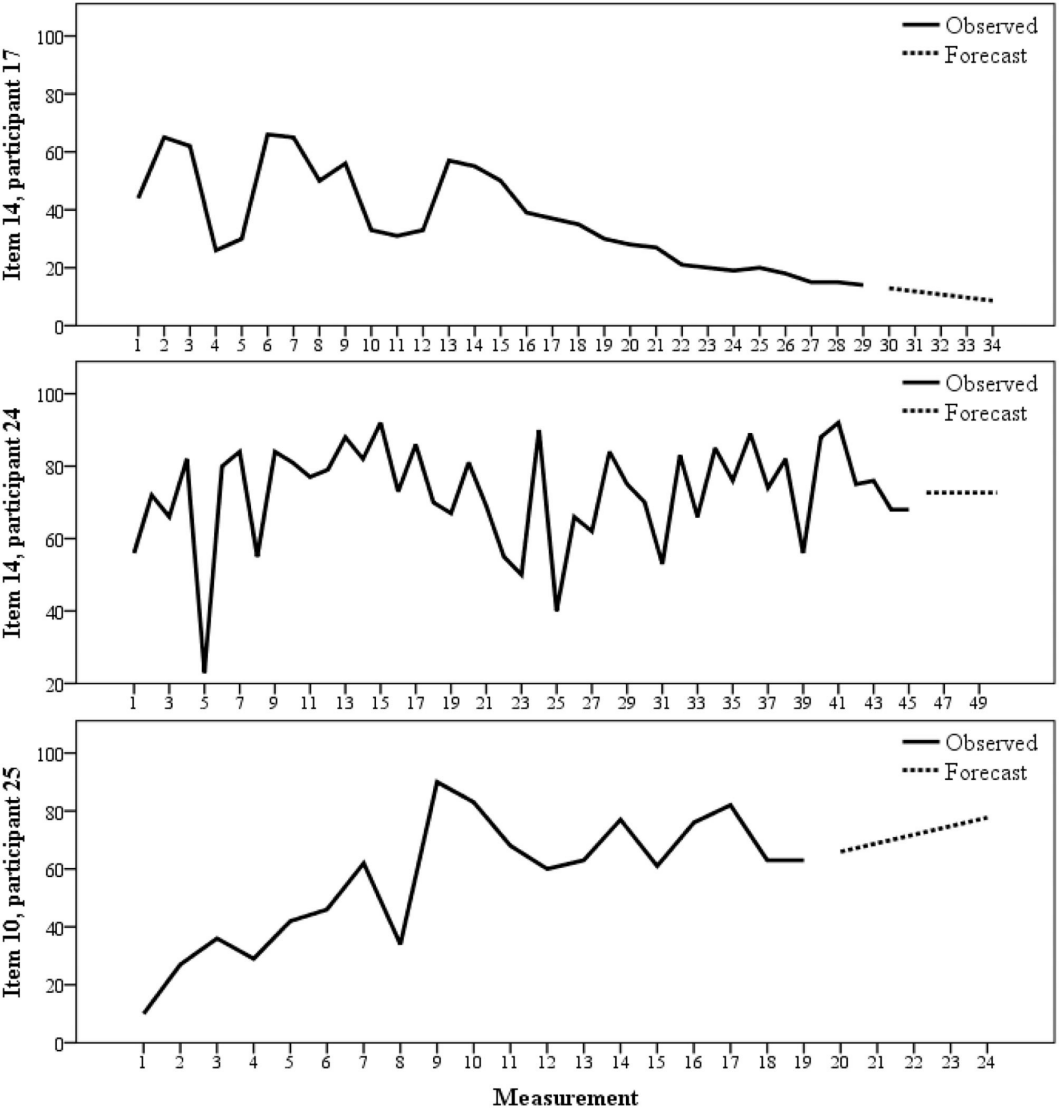
PSIXPORT observed and predicted scores with ARIMA models in three participants.

Figure 1 depicts three examples of observed scores, and forecasts obtained via ARIMA models. These examples, chosen from three different participants and two distinct items which were selected due to they clearly depict forecasts with upward, horizontal, and downward trends, respectively, based on previous values.

## Discussion

The present study assessed the Psixport mobile app feasibility to collect real-time data about injured athletes' psychological responses (cognitive appraisals, emotional responses, behavioral responses, and pain perception) during their rehabilitation process, to test the app's usability for subjects, and check if gathering information by multiple assessments from EMA approach provides more accurate information about injured athletes' thoughts, emotions, and behaviors than one-time retrospective reports.

Regarding Psixport's feasibility to collect real-time data, the app's compliance ratio (55.7%) was similar to other mHealth apps compliance ratio used in previous studies where subjects were assessed during periods longer than 10 days (Mulvaney et al., 2012; Könen et al., 2015, 2016). For instance, Mulvaney et al. (2012), assessing diabetes adherence patterns in adolescents, got 59% EMA's response rate for a 10-day calling period, and Könen et al. (2015, 2016) assessing sleeping behaviors consequences on childhood, founded similar surveys' compliance ratios depending on the daytime where surveys were prompt, ranging from 57% (afternoon) to 66% (morning). Han et al. (2018) reported a 49.29% response rate for the mHealth app designed to gather information about drug use through daily assessments during a 4-week assessment period. Therefore, we can consider Psixport's user engagement in line with what similar apps have obtained in previous studies.

App's compliance over the time was good according to the fact that the average number of evaluations completed per athlete was higher (*M* = 22.2) than the minimum of evaluations initially requested (15). However, this commitment varied widely through subjects. Some subjects completed daily assessments almost four times more than the minimum requested. These results lead us to confirm that the time length the record was required did not suppose an obstacle for subjects to commit with the app.

Psixport's response ratio decreased over time, which is a phenomenon that has been reported on previous mHealth EMA approach app's studies too (Kenny et al., 2016). Heron et al. (2017) conducted a systematic review of Mobile-Technology-based EMA methods which analyzed 24 studies and reported that, in 23 of them, the number of days of EMA data collection ranged from 4 to 31, in line with Psixport's number of evaluations requested (15). Moreover, Heron et al. (2017) set that research question must be considered to establish EMA surveys' frequency and duration.

Concerning the second goal of the study, the app's usability according to engagement, functionality, aesthetics, and information, as well as subjective quality has shown to be fairly satisfying. Comparing Psixport's scores with other mHealth apps scores assessed with the original MARS test (Bardus et al., 2016), Psixport gathers higher scores in all domains; engagement, functionality, aesthetics, information, and total score.

The presence of features and techniques such as semi-automated tracking (self-monitoring) inside the apps were associated with higher app quality scores in MARS in previous research, specifically for engagement, functionality, and aesthetics (Bardus et al., 2016). The high scores obtained by Psixport in those aspects could be explained due to the app included a “feedback” module that allowed athletes to self-monitoring their progress during the rehabilitation process. Psixport included prompt notifications as well, which has been also associated with high scores for app quality in previous research (Bardus et al., 2016).

Moreover, this app has demonstrated Good/Excellent levels of functionality and information for subjects, becoming the strong characteristics of the app for users. This could be due to Psixport is a user-friendly app. Only two subjects asked some doubt related to the app functioning and no one reported questions regarding items' understanding or the answering method. In line with this, perceived information quality provided by mHealth apps has been associated with a specific techniques' combination such as feedback and self-monitoring (Bardus et al., 2016), both included in Psixport, which could explain the high scores obtained by the app too.

Respecting the app's subjective quality and perceived impact scores assessed by items “*Would you recommend this app to people who might benefit from it?”* and “*Would you pay for this app?”* the results show an Acceptable/Good assessment from users, being most of the participants willing to recommend the app use for future injured athletes though not paying for it. These athletes' good appreciations about app usefulness may be related to the feedback the app provided, which made them aware of their own evolution in their rehabilitation process.

Finally, considering Psixport total score in uMARS and taking into account previous research conclusions obtained by accessing multiple mHealth apps with original MARS (Bardus et al., 2016), we can suggest that Psixport could be considered as a high-quality app.

Our third goal was to compare the differences between EMA data gathered using the app and the data gathered by a one-time retrospective method. The comparison between means of Psixport's averaged and forecasted data and retrospective data revealed that the app gathers more accurate information about athletes' cognitive appraisals, emotional responses, and pain perception than the one-time retrospective method. Higher correlations were found between EMA averaged data gathered with Psixport and EMA forecasted data compared to correlations between those two and one-time retrospective data gathered, supporting our initial hypothesis and previous research, which found that multiple assessments across time such as diary-based methodologies could gather accurate information about subjects' behaviors and experiences than retrospective reports (Reis and Wheeler, 1991).

For emotional responses, positive correlations between averaged—forecasted scores were also found, particularly for the emotional valence dimension (Happy–Sad). These results are aligned with previous research findings, which concluded that subjective experiences such as the intensity of feelings are difficult to be accurately assessed by retrospective methods because these experiences are poorly represented in the memory (Robinson and Clore, 2002).

Similarly, positive correlations were found between averaged—forecasted scores for three cognitive appraisals' dimensions (devastation, feeling cheated, and isolation), and maximum pain dimension, but not between averaged and forecasted scores with retrospective scores. This could be explained by Schwarz's (2007) conclusions where he set that subjects' retrospective reports about their past behaviors and experiences were similar to their present behavior or experience at the time of the assessment. Moreover, Eich et al. (1985) also found similar results regarding subjects' pain perceptions.

However, positive correlations between averaged and forecasted scores were not found for all cognitive appraisals, emotional responses, and pain perception dimensions assessed. This could be explained due to gathering real-time data can reduce the influence of biases related to retrospective reports (Schwarz, 2007), but does not eliminate other kinds of self-reports biases such as problems on question comprehension, or the order in which responses alternatives or questions are presented (Schwarz, 1999; Sudman et al., 2004).

Therefore, we can conclude that gather information about changing and dynamic variables such as injured athletes' cognitive appraisals, emotional responses, or pain perceptions through multiple assessments across the time may provide more accurate information than the one-time assessment approaches.

## Strengths and Limitations

There are some limitations to this study. Firstly, the convenience sample and its small size can limit the generalization of the results obtained. Nevertheless, Heron et al. (2017) reported population size ranged from 6 to 303 in the EMA studies included in their systematic review, which shows that EMA studies' sample sizes are quite variable.

Secondly, we have obtained a response rate which is clearly in line with the compliance ratio found in previous studies where the subjects were assessed during periods longer than 10 days. Nevertheless, there is room to an improvement of the commitment to complete the assessment. Therefore, we think gamification might contribute to improve participation and keep participants committed to the task. In the current form of the app, we included a feedback module associated to the collection of different points. We know that PSIXPORT is far from being a gamified app. However, we are currently working on incorporating more aspects of gamification. Based on Plass et al. (2015) model, future versions of the app will include different levels or phases, a progress bar, more badges, and a public ranking of response ratio, as well as trying to revamp the visual aesthetics to foster the commitment with the task.

Thirdly, there should be noted that perception of usability was measured using the uMARS. This test was designed to assess users' perceptions about the mHealth apps used. Psixport cannot be fully considered as a mHealth app due to the app's main goal is to gather real-time information about injured athletes avoiding any kind of intervention across their rehabilitation processes. However, uMARS main focus is to assess apps' engagement, functionality, aesthetics, and information quality Stoyanov et al. (2016), not intervention effectiveness.

## Conclusions

In conclusion, we can set that Psixport could be considered as a reliable and appropriate tool for gathering real-time information about injured athletes' behaviors, cognitive appraisals, emotional responses, and pain perceptions.

This app could be used to register athletes' thoughts, emotions and behaviors, and their changes, across their rehabilitation process. This can help therapists to design specific rehabilitation programs adapted to athletes' needs and progress, which could increase the likelihood of their effectiveness.

## Data Availability Statement

The original contributions presented in the study are included in the article/supplementary material, further inquiries can be directed to the corresponding author/s.

## Ethics Statement

This study was reviewed and approved by the Universidad Autonoma de Madrid's IRB. All participants provided their written informed consent to participate in this study.

## Author Contributions

All authors listed have made a substantial, direct and intellectual contribution to the work, and approved it for publication.

## Conflict of Interest

The authors declare that the research was conducted in the absence of any commercial or financial relationships that could be construed as a potential conflict of interest.

## Publisher's Note

All claims expressed in this article are solely those of the authors and do not necessarily represent those of their affiliated organizations, or those of the publisher, the editors and the reviewers. Any product that may be evaluated in this article, or claim that may be made by its manufacturer, is not guaranteed or endorsed by the publisher.

## Appendix

### Retrospective Questionnaire

*Please, move and place the bar where fits for each one of the items below based on what happened to you during the rehabilitation process:*
